# Physical Activity in High School Classrooms: A Promising Avenue for Future Research

**DOI:** 10.3390/ijerph19020688

**Published:** 2022-01-08

**Authors:** Barbara Fenesi, Jeffrey D. Graham, Madeline Crichton, Michelle Ogrodnik, Jasmyn Skinner

**Affiliations:** 1 Faculty of Education, Western University, London, ON N6G 1G7, Canada; mcricht3@uwo.ca (M.C.); jskinn7@uwo.ca (J.S.); 2 Faculty of Health Sciences, Ontario Tech University, Oshawa, ON L1G 0C5, Canada; jeffrey.graham@ontariotechu.ca; 3 Department of Kinesiology, McMaster University, Hamilton, ON L8S 4L8, Canada; ogrodnm@mcmaster.ca

**Keywords:** classroom-based physical activity, physically active classrooms, secondary schools, adolescent well-being, physical activity, academic achievement

## Abstract

Adolescence represents a sensitive period whereby lifestyle factors such as physical activity can have profound, long-lasting effects on development and later life habits. However, adolescence constitutes a period of frequent sedentary behaviour. Among children, integrating physical activity into elementary school classrooms has been shown to reduce sedentary behaviour and improve academic achievement and overall physical and mental health. However, this promising area of research has not extended to adolescents and high school classrooms. In this paper, we describe the benefits of conducting research on the impact of physically active high school classrooms, and highlight the challenges and potential misconceptions associated with research in this field. Specifically, we review research on the role of physical activity in adolescent development, the benefits of classroom-based physical activity for children, and discuss the factors that may have led researchers to focus on classroom-based physical activity primarily for children, despite the potentially similar benefits for adolescents.

## 1. Introduction

The last several decades have seen a rapid growth of interest in elucidating the relationship between physical activity and cognitive functioning in children (3–11 years of age), adolescents (12–18 years of age) and older adults (65 years of age) [1,2]. One of the main reasons for this interest is because of the overwhelming evidence showing a positive relationship between physical activity, brain functioning and cognitive performance across the lifespan [3,4,5,6,7,8,9,10]. Given the growing rate of sedentary behaviour among children in many parts of the world [11], and the corresponding consequences for children’s well-being, many researchers and policy makers have turned to classrooms as potential avenues to increase children’s physical activity behaviour. More specifically, a great deal of research has focused on evaluating how integrating physical activity into elementary school classrooms can reduce sedentary behaviour and improve academic achievement and overall well-being among children [12,13,14,15,16]. However, this work has not extended to high school classrooms, despite adolescents constituting one of the most sedentary portions of the population [11], and ample evidence demonstrating the critical role that physical activity plays in supporting healthy adolescent development [10,17]. Research must be extended to high school settings so that adolescents can begin to have access to evidence-based physical activity interventions in their classrooms.

In this paper, we aim to describe the potential benefits of conducting research on the impact of physically active high school classrooms and to highlight the challenges and possible misconceptions related to conducting this research. The following paper discusses (1) the role of physical activity in supporting adolescent neurocognitive and psycho-emotional development, (2) the benefits of classroom-based physical activity for children in elementary school and the promising translation to adolescents in high school, and (3) the potential factors that have led researchers to focus on the benefits of classroom-based physical activity mainly for children, despite potentially similar benefits for adolescents.

## 2. The Role of Physical Activity in Supporting Adolescent Neurocognitive and Psycho-Emotional Development

Significant developmental changes in brain regions associated with higher order cognitive functions (e.g., problem solving, sustained attention, working memory, inhibitory control) occur during childhood [18,19]. However, these developmental changes continue throughout adolescence, with some structures and functions undergoing the most significant changes during this time [20]. Specifically, adolescence is characterized by substantial increases in white matter density throughout the brain, which supports the smooth flow of information [21]. There is also a corresponding decrease in grey matter density; this is thought to be attributed to synaptic pruning, which is an essential process that helps to selectively strengthen frequently used connections, aiding in efficient brain functioning as adolescents learn new complex information [20,21].

Engaging in regular physical activity at any age enhances new blood vessel formation and extension in the brain, as well as increases neurotrophic factors that support neuronal birth and proliferation [10,17]. Specifically, the levels of Brain-Derived Neurotropic Factor (BDNF), a protein associated with changes in learning, memory, mood, and anxiety, have been found to increase in response to exercise due to increased production of the component molecules which make up the BDNF protein [22,23]. Given the rapid pruning and myelination during adolescence, the neural mechanisms reinforced through physical activity can support the fine-tuning of cognitive functions that promote learning and memory processes. Physical activity also has immediate direct effects on the adolescent brain including elevating mean cerebral blood flow and increasing plasma levels of key neurotransmitters related to heightened arousal, all of which may support enhanced cognitive functioning [24,25].

Beyond the neurocognitive benefits, physical activity also has profound benefits for psycho-emotional outcomes among preschoolers, children, and adolescents [26]. With a specific focus on adolescents, those who engage in more physical activity than their peers have reported experiencing less anxiety, depression, and other mood-related disturbances; they also reported higher ratings of self-esteem and self-concept [27,28,29,30]. These findings are especially relevant for adolescents as many mental health challenges emerge during this developmental period [31,32]. Psycho-emotional states during adolescence can have long-term consequences for later life health, with some research showing that low self-esteem in adolescence can lead to poor health outcomes in adulthood [33]. The adolescent period also plays an especially important role in setting up positive physical activity habits that become the foundation for later life habits [34]. In a large-scale review of tracking studies across the world, participation in physical activity from adolescence to adulthood was shown to be stable, or “tracked” over time [35,36]. This means that highly active adolescents are more likely to become highly active adults, and vice versa; inactive adolescents are more likely to become inactive adults.

Despite the positive effects of physical activity on adolescent neurocognitive and psycho-emotional development, they are one of the least active age groups. On a global scale, the prevalence of physical inactivity in adolescence has been reported at an alarming 80% [11]. In the United States, physical activity levels decline as children reach adolescence, with 6–11 year olds spending approximately 88 min per day engaged in moderate to vigorous physical activity, compared to only 33 min per day for 12–15 year olds, and 26 min per day for 16–19 year olds [37]. These figures fall far below the 24 h movement behaviour guidelines from the World Health Organization, which recommends 60 min per day of moderate to vigorous physical activity [14,38]. In Canada, boys between the ages of 6–10 years old have been reported to be meeting the WHO’s recommended guidelines [39]. However, between the ages of 11 and 19, boys no longer meet the recommended guidelines. Girls fail to meet the minimum requirements in all age groups, with physical activity participation decreasing from 56 min to 38 min between the ages of 10 and 19. The most recent ParticipACTION Report Card gave Canada an “F” grade for 24 h movement behaviours, with less than 20% of children and adolescents meeting the recommended WHO guidelines [40]. Relatedly, in both Canada [39] and parts of England [41], sedentary time during waking hours was found to increase from about 51% at 7 years of age to between 62 and 74% at 15 years of age. As previously mentioned, physical activity participation has the strongest tracking record from adolescence to adulthood [35,36]. This means that adolescence is a critical window whereby exposure to and encouragement for physical activity engagement can positively affect adulthood participation. Taken together, these findings underscore the importance of increasing physical activity behaviour in adolescence not only to support neurocognitive and psychological development, but also to increase the likelihood of later life engagement in a physically active lifestyle.

## 3. The Benefits of Classroom-Based Physical Activity for Children in Elementary Schools and the Promising Translation to Adolescents in High Schools

Opportunities for children to engage in regular physical activity have been on the decline for many years, with cuts to after school programs and physical education resources across many parts of North America [42,43,44,45,46]. Given that youth spend the majority of their day in school [12,40], a logical strategy for increasing levels of physical activity for children has been incorporating physical activity directly into classroom instruction. This alleviates some of the exclusive reliance on after school programs and physical education classes to provide children with daily activity. Increasing classroom-based physical activity can also help reduce inequitable access to physical activity opportunities for children and youth from low socioeconomic status backgrounds, given that extra-curricular activities can be quite expensive and demanding of parental time [47,48].

Classroom-based physical activity has gained significant attention over the last decade or so, with recent neuroscientific findings placing emphasis on the strong link between movement and learning [14]. Classroom-based physical activity can include active breaks, teacher-led active lessons that integrate academic content, or the use of standing desks [12,49,50]. Some examples of these practices include a three-to-five-minute dance break between lessons, instructing students to answer math questions by performing a certain number of physical movements (e.g., jumping jacks) [51], learning multiplication tables by doing “invisible jump rope”, performing two-part muscle contraction movements to better understand how two words become a contracted word, and many others [52]. Several recent systematic reviews have found that elementary classrooms that are physically active support greater academic achievement compared to traditional sedentary elementary classrooms [12,13,15]. Children who participate in classroom-based physical activity are also more likely to meet the WHO’s recommendation of 60 min of daily physical activity [53]. Classroom-based physical activity has also been shown to increase students’ feelings of joy and motivation to learn, as well as classroom behaviour (i.e., time-on-task) and various aspects of academic achievement, with these effects occurring both acutely and over the long-term [12,16,54,55]. Additionally, qualitative studies of both high school students and high school teachers support an interest and willingness to engage in physical activity in the classroom [56,57,58]. However, there is currently no evidence of research investigating the impact of physical activity in high school classrooms (beyond physical education settings) on adolescent cognitive and academic outcomes.

Several recent systematic reviews highlight the lack of research investigating the impact of physical activity in high school classrooms on adolescent cognitive and academic functioning. For instance, Masini et al. (2019) showed that of 22 studies examining the impact of classroom-based physical activity breaks (i.e., active breaks spaced in between the delivery of academic content), all studies occurred in elementary schools [15]. Similarly, Bedard et al. (2019) showed of the 25 studies examining the impact of classroom-based physically active lessons (i.e., active breaks that include the delivery of academic content), none of the studies occurred in high schools [12]. In addition, our own thorough search of the literature did not reveal any studies involving physically active classroom interventions at the high school level since the above reviews were conducted.

Although parts of Canada have instituted the Daily Physical Activity (DPA) Policy, which requires at least 20–30 min of instructional time during the school day be devoted to physical activity per day (outside regular physical education hours or recess), the policy only targets classrooms up until Grade 8 or 9 (i.e., individuals aged 13–15 years). This is the case for 9 out of the 13 provinces and territories in Canada, with only 4 recognizing the value of DPA for student success up until Grade 12 [59]. In 2008, the U.S. Department of Health and Human Services recommended that elementary and high school classrooms be used to increase physical activity and address child and youth obesity [60]. One of the many reasons for this recommendation was that only 30% of adolescents reported attending physical education class daily while in school, with many students viewing physical education as non-essential to their academic success. Furthermore, in 2017 in the United States, only 26% of high school students indicated that they participated in at least 60 min of physical activity per day during the week [60]. Given the insufficient engagement in regular physical activity among adolescents, it is evident that integrating physical activity directly into regular high school classrooms throughout the school day would also be an ideal avenue to promote adolescent physical activity behaviour. However, the research community has mainly focused its efforts on identifying successful strategies and programs for the implementation of physical activity in elementary school classrooms. Therefore, the last section of this paper will discuss potential reasons why researchers have not investigated the impact of physical activity in high school classrooms, as well as offer some solutions on how to move the field forward.

## 4. Factors That Have Led Researchers to Focus on the Benefits of Classroom-Based Physical Activity Mainly for Children Rather Than Adolescents

The first reason may be that although many researchers and teachers recognize the importance of physical activity for various aspects of adolescent cognitive and physical health, there are numerous environmental, experiential, and systemic barriers that interfere with classroom implementation, despite informed views and good intentions. In a recent study, high school science teachers expressed interest and willingness to implement physical activity within their classrooms and believed that it would help keep students engaged [58]. However, they found lack of space in the classroom, concerns about behaviour management, and lack of knowledge to be substantial barriers to implementation. Several of these teachers expressed a need for more information about strategies for implementing physical activity with adolescents specifically. Prior studies involving high school teachers of Native American students found similar patterns with teachers expressing interest in incorporating physical activity into their classroom, but identifying scheduling and classroom management as barriers to implementation [44,61]. Although these studies contain small samples and may have limited generalizability, they demonstrate a substantial interest from high school teachers in utilizing classroom physical activity, but also identify a lack of knowledge and physical space as key barriers. Research in this domain would likely be well-received by educators and could help them implement physical activity into their classrooms more confidently and effectively.

In addition to physical space, the daily structure of high school classrooms may also be less amenable to physical activity compared to elementary classrooms. In high schools, classes are subject-specific, and each class is taught by a different teacher. In elementary schools, students are typically in the same room with the same teacher all or most of the day. This means that the high school setting may present additional logistical challenges for researchers as schedules are less flexible and educators may be less willing to spend time on research activities when they only have each group of students for a limited time each day. A common design in elementary research has been to allow teachers to implement physical activity interventions whenever they prefer (i.e., simply asking teachers to do one ten-minute activity per day) [62,63]. However, other research in elementary schools has consistently occurred within a single class period [64,65]. Thus, a single class period can also feasibly be used to study classroom physical activity interventions at the high school level. Beyond logistical challenges, the academic content demands in high school are different from elementary school, in that the coursework requires higher levels of thinking and reasoning and more synthesis of information. This may be more difficult to integrate with physical activity than elementary level coursework that relies more on repetition and lower-order cognitive skills. However, the differences between high school and elementary school make it even more important to study physical activity at the high school level. The nuances of changing classrooms and teachers throughout the day, as well as the unique needs and greater complexity of each content area, provide ample unanswered research questions. For example, how should physical activity be implemented differently in a math class compared to a chemistry or history or language arts class? What strategies can different teachers use for coordinating the implementation of physical activity for students throughout the day? What type of physical activity is best-suited to support the learning of higher-order concepts? Should researchers pilot full-year hybrid courses to determine what academic content may be best suited for a physical activity setting? Although the logistical challenges of studying high school physical activity certainly exist, they should not deter researchers from doing work in this environment, especially considering the clear and unique benefits of investigating these questions.

The second reason why researchers may not have focused on how classroom-based physical activity similarly benefits high school students may be because findings on the effects of a single bout of physical activity on adolescent cognitive functioning are mixed. A meta-analysis by Ludyga et al. (2016) found that adolescents showed no or only small improvement in cognitive functioning after a single physical activity session [66]. However, an earlier meta-analysis by Chang et al. (2012) found the opposite; adolescents showed the most pronounced cognitive improvements compared to children and older adults [67]. Yet, others have shown that irrespective of developmental age, a single bout of physical activity can promote cognitive functioning [10]. More recent work has shown that single physical activity sessions, irrespective of intensity, support cognitive inhibition among adolescents [68]. Discrepant findings surrounding the benefits of short bouts of physical activity on adolescent cognitive function may discourage researchers from investigating how short bouts of classroom-based physical activity can support adolescent cognitive and academic success. While mixed results are often commonplace in scientific research, the evidence supporting the benefits of short physical activity bouts on children’s cognitive functioning is more consistent [7,10]. Researchers may therefore have been more comfortable translating the application of short bouts of physical activity from the lab to the elementary school classroom. It is important to note however that the longitudinal data showing the long-term benefits of physical activity for adolescent neurocognitive and psychological outcomes are more consistent [69,70,71]. Thus, even if short bouts of classroom-based physical activity are not as effective for promoting cognitive functioning among adolescents when compared to children, the accumulation of repeated bouts of physical activity each day, across time, would still likely yield long-term adaptations with positive cognitive and psychological results [72].

The third reason why research on high school physical activity interventions is underrepresented may reflect a lingering misconception that significant developmental changes do not continue throughout adolescence and into adulthood. Many researchers suggest that adolescents and adults may be less responsive to physical activity interventions because their cognitive capacity is at “ceiling”, and therefore, less likely to be affected by environmental input [66]. According to the inverted-U shape hypothesis [73,74,75], children and older adults are on the tail ends of the inverted-U spectrum and, thus, have the most room for upwards mobility; consequently, they are the most likely to reap neurocognitive benefits from behavioural interventions such as physical activity [66]. However, it is undeniable that adolescence is characterized by dramatic changes in cognitive and psychosocial functioning [21]. Adolescents become more self-aware and self-reflective than prepubescent children, develop a capacity to hold in mind more multidimensional concepts, and are able to think more strategically. The roots of these shifts are anatomical and functional brain changes that occur during adolescence. The most significant changes occur in the prefrontal cortex—the region of the brain that supports higher order cognitive functioning including attention regulation, inhibitory control, working memory and problem solving [20]. Whereas sensory and motor brain regions become fully myelinated in the first few years of life, neuronal axons in the prefrontal cortex continue to be myelinated well into adolescence. Myelination allows for quicker transmission of information and is essential for more complex brain processes to occur. Synaptic elimination (or pruning) also dramatically increases during adolescence, leading to more efficient neural network communication [20,21]. Clearly, the adolescent brain is in a sensitive period and is amenable to lifestyle factors such as physical activity having a positive impact on its structure and function. Given the previously described impact of physical activity on the cellular and metabolic structures of the brain [10,17,25], there is tremendous promise for the use of classroom-based physical activity to upregulate engagement in physical activity behaviour throughout the day and, consequently, to support adolescent neurocognitive development.

The fourth reason why researchers may not have focused on the role of physical activity in the high school classroom may be because of the systemic messaging by many educational systems that physical activity is less relevant as you enter adulthood. Physical education class is not mandatory in many school curricula in the United States and Canada beyond grade nine [76,77]. After that point, adolescents are encouraged to focus their attention on specific academic and practical domains that they wish to explore as potential careers after high school. Additionally, the focus at the high school level (in some countries) turns to high-stakes assessment and post-secondary admissions [78,79]. This is reported to lead to a greater focus on content areas being tested, and less time spent on other activities [80,81]. This change in focus could reduce already limited time to devote to classroom-based physical activity and constrain pedagogy to assessment-focused methods. While the focus on academic content is logical in a school setting and an important preparation for students entering adulthood, it should not come at the expense of maintaining healthy habits such as participating in regular physical activity, especially since physical activity participation can directly support academic success and overall physical and mental well-being [7,14]. Furthermore, as previously stated, adolescents who remain physically active are significantly more likely to become physically active adults [33,34]. If the ultimate goal of schools is to produce cognitively healthy and academically adept individuals, ignoring the critical value that physical activity plays in that process will make that goal much more challenging to achieve [14]. Our society’s brain–body disconnect is certainly not a new concept, dating all the way back to 17th century Cartesian dualism [82]; it is also an issue that spans well beyond the scope of educational systems, and while an equally important topic of conversation, extends beyond the scope of the current paper. However, by considering the irrational demand for young students (and many working professionals) to sit at a desk or computer for 6+ hours a day and produce cognitive output, while any movement or physical activity is often seen as off-task behaviour, we can see that a cultural shift is needed. Researchers have the opportunity to begin shifting the culture by expanding our scope of classroom-based physical activity research to include the adolescent population within their high school setting.

Lastly, a fifth potential reason why researchers may have steered away from investigating how classroom-based physical activity can support adolescent success is potentially due to the change in attitudes and increase in self-consciousness during adolescence. Teens begin to place more value on the opinions of their peers, are going through bodily changes due to puberty, and are in a sensitive period of self-esteem development [1,21]. Adolescence is a time of biological and psychological upheaval. Incorporating bouts of physical activity into the classroom may be met with resistance for a multitude of reasons including discomfort with one’s own changing body, fear of social evaluation and judgment, and perhaps a newfound questioning of authority and rules. The general temperament of the classroom will also likely influence the receptivity towards physical activity; if a majority (or even a vocal minority) of students view classroom-based physical activity as “un-cool”, the risk of social ostracization will almost always outweigh the willingness to participate. Indeed, adolescent girls indicated that they would not be willing to participate in dance breaks or other “silly” physical activities during class. However, the same girls suggested that they would be more comfortable with incidental or content-related physical activity and thought it would be beneficial for their learning [56]. It is also possible that the format of classroom-based physical activity (i.e., it takes place in short bouts and is non-competitive) may not fit with adolescent preferences for physical activity [83]. Some adolescents may prefer competitive and athletically demanding physical activity [83,84], and thus may have minimal enthusiasm for classroom-based physical activity. In general, adolescents are more motivated to engage in activities they choose themselves [84,85] While there are still some benefits to just standing up and moving around, motivation and engagement during classroom-based physical activity is an important contributor to its efficacy [86,87]. As a result, researchers may have viewed the high school classroom as a futile place to incorporate physical activity. However, several researchers argue that the plethora of biological and psychological changes during adolescence are all the more reason to incorporate more physical activity into the school day [88,89]. Specifically, given the role of physical activity in decreasing anxiety and increasing self-esteem, greater exposure to physical activity throughout the school day may actually help mitigate those burgeoning issues. Furthermore, if classroom-based physical activity becomes a more commonplace experience for students, extending from elementary school to high school, anxieties around participation may be reduced given the removal of novelty. If classroom-based physical activity can become normalized for children and adolescents, participation would be less daunting and would be freer of social stakes. Adolescents would then be able to reap the many neurocognitive and psycho-emotional benefits of daily physical activity.

## 5. Future Directions and Conclusions

Adolescence represents a sensitive period whereby lifestyle factors such as physical activity can have profound, long-lasting effects on development and later life habits. However, there is an absence of research examining the impact of classroom-based physical activity in high school settings. As researchers in the field ourselves, having mainly focused on younger and older age groups, our goal was not to condemn others for not extending their classroom-based research to adolescents. Rather, the goal was to begin a conversation about how to translate the excellent research that has been carried out with elementary school children to high school adolescents. Some high schools in Ontario (Canada) have begun incorporating physical activity into classrooms, especially mathematics. However, this work is being led only in specific schools and classrooms by teacher champions who are well-informed of the link between physical activity and academic learning. One way to begin moving this field forward is for researchers to work with these existing classrooms and empirically evaluate their efficacy using cognitive and psycho-emotional assessments, as well as learning outcomes. This will begin to offer much-needed evidence on the effects of classroom-based physical activity on adolescent cognitive and psycho-emotional functioning. In addition, qualitative research using surveys and focus groups involving high school teachers and administrators could be used to better understand barriers to implementation. This information could then be used to inform the development of effective classroom-based programs in high schools. There is a mixture of social, cultural, academic, and institutional-level factors that need to be identified and addressed in order to promote greater research on the role of classroom-based physical activity in adolescent academic, cognitive, and psycho-emotional well-being.

This paper aimed to describe the benefits of conducting research on the impact of physically active high school classrooms and bring to light the potential reasons why physical activity interventions in high school classrooms are underrepresented in research. It also aimed to highlight the research demonstrating the vast neurobiological changes occurring in the adolescent brain and in their behaviour, and to underscore the receptivity that this sensitive period offers for physical activity to support the critical changes taking place during that time. We hope that this discussion encourages more classroom-based research in this area so that the developing adolescent brain and body can also benefit from routinized and consistent physical activity engagement throughout the school day.

## Data Availability

Data sharing not applicable.
